# Bladder-Sparing Therapies for Bacillus Calmette-Guérin-Unresponsive Carcinoma In Situ: An Upper and a Lower Response Group With an Unstable Boundary

**DOI:** 10.7759/cureus.117332

**Published:** 2026-10-01

**Authors:** Ryo Kasahara

**Affiliations:** 1 Urology, Japanese Red Cross Hadano Hospital, Hadano, JPN

**Keywords:** bayes theorem, bcg-unresponsive nmibc, bcg vaccine, bladder-sparing therapy, specification curve analysis, urinary bladder neoplasm, urothelial carcinoma

## Abstract

Objectives: The objectives of this study are to determine which orderings among five bladder-sparing therapies for Bacillus Calmette-Guérin (BCG)-unresponsive carcinoma in situ are supported by the published response data, and which depend on how response is defined and analysed.

Methods: The pivotal cohorts of the five regimens approved or in rolling application were identified, and counts derived from the published data were analysed with independent binomial models and Beta(1,1) priors. Papillary-only cohorts were not eligible. Complete response, as each trial defined it, and a 12-month response-related quantity were examined, with all comparisons repeated across 10 specifications of the response definition, the analysis population and the anchor.

Results: Five cohorts of 82 to 110 patients were included. Complete response estimates were 81.9%, 75.1%, 70.4%, 53.4% and 40.8% for TAR-200, cretostimogene, nogapendekin alfa inbakicept plus bacillus Calmette-Guérin (NAI plus BCG), nadofaragene firadenovec and pembrolizumab. The corresponding 12-month quantities were 45.9%, 46.4%, 45.2%, 24.6% and 19.2%, which differ in anchor, population and construct. All six comparisons between the higher three and the lower two excluded zero by 20.5 to 27.2 percentage points, whereas none within the higher three did so under any 12-month specification. Of the 100 comparisons, 44 included zero, and no comparison excluded zero in the opposite direction. Two of the 10 specifications depend on a three-month cretostimogene count that has not been published and was bounded rather than observed. Restricting the analysis to the four approved agents, excluding cretostimogene, left these findings unchanged.

Conclusions: The published response quantities form an upper and a lower group rather than a stable ranking of five. TAR-200 and cretostimogene remained above the lower two agents in 8 to 10 specifications, whereas the separation of NAI plus BCG was less stable. Within the upper group, the agents could not be ordered.

## Introduction

Radical cystectomy is the guideline-recommended treatment for high-risk non-muscle-invasive bladder cancer (NMIBC) that does not respond to Bacillus Calmette-Guérin (BCG) [1]. Many patients are unfit for it or refuse it. For them, the US Food and Drug Administration (FDA) defined BCG-unresponsive disease and accepted single-arm trials with complete response (CR) as the basis for approval [2]. Five agents have reached approval or rolling submission by this route: pembrolizumab [3], nadofaragene firadenovec [4], nogapendekin alfa inbakicept (NAI) plus BCG [5], TAR-200 [6], and cretostimogene grenadenorepvec [7]. Each is supported by a pivotal cohort of patients with carcinoma in situ (CIS), with or without papillary tumours.

The distinction from papillary-only disease matters. In CIS, measurable disease is present when treatment begins, so the endpoint is CR. In papillary-only disease, all visible tumours are resected first, so the endpoint is disease-free survival. The two are not interchangeable, and the papillary-only cohorts of these trials are outside the scope of this analysis [8]. No ongoing head-to-head trial between any two agents was found.

CR rates of about 41% to 82% are therefore placed side by side in reviews and congress presentations, and guidelines list multiple options without stating a preference [1]. Published syntheses and commentaries have similarly juxtaposed bladder-sparing regimens across trials [9,10]. What has not been quantified is how far the apparent ordering changes when reasonable alternative response definitions, analysis populations, anchors and re-induction rules are applied to the same pivotal CIS cohorts. This study asked a narrower question than which agent is most effective. Which statements about the ranking are supported, and which depend on how response is defined and analysed?

## Materials and methods

Design and reporting

This is a Bayesian re-analysis of response counts derived from the published summary data of five trials [3-7]. Trial identification and selection used the search and eligibility criteria described below. Each cohort was analysed on its own; no pooling was performed.

The study was not prospectively registered, and one investigator (RK) performed screening, extraction and analysis without independent duplicate checking. Every count entered into the analysis was verified against the primary report, and the two re-expressed counts (Table 1) were recomputed from the published numerators and denominators; the specification counts (Table 2) were checked against the primary reports a second time before analysis. Extraction preceded protocol finalisation, so prospective registration was no longer possible. Several analyses were added after the comparative outputs had been calculated and are therefore exploratory. These are the specification grid, the present form of the first-induction restriction, the alternative 12-month substitutions, the tipping-point analysis, the sceptical priors and, added during peer review, the analysis restricted to the approved agents. A simulation of between-cohort error, an analysis in which every denominator was increased, and the full prior-sensitivity results were also performed; they are not reported here and are available from the author.

**Table 1:** Characteristics of the five included cohorts Primary trial reports: KEYNOTE-057 [3], the phase 3 nadofaragene trial [4], QUILT-3.032 [5], SunRISe-1 [6], and BOND-003 [7]. ᵃ All observed CRs occurred within three months of the first dose. ᵇ Central review of 77 assessable patients reported separately [11]. ᶜ Among responders. ᵈ The FDA review reports 28 of 48 responders maintaining CR for at least 12 months; re-expressed on the 77-patient FDA efficacy population this is 28/77 (36.4%), used in specifications 8 and 9 [11]. ᵉ Definitions, reporting periods and denominators differ between trials; these figures are not directly comparable. CR: complete response; NAI: nogapendekin alfa inbakicept; BCG: Bacillus Calmette-Guérin; FDA: US Food and Drug Administration; BLA: biologics licence application; NR: not reported.

Characteristic	KEYNOTE-057 (pembrolizumab) [3]	Phase 3 (nadofaragene) [4]	QUILT-3.032 (NAI + BCG) [5]	SunRISe-1 Cohort 2 (TAR-200) [6]	BOND-003 Cohort C (cretostimogene) [7]
Phase	2	3	2/3	2b	3
Regulatory status (10 Aug 2026)	Approved 2020	Approved 2022	Approved 2024	Approved 2025	Rolling BLA initiated Nov 2025; completion expected Q4 2026
Patients analysed for efficacy	96	103	82	85	110
Concomitant papillary disease	NR	NR	NR	32.9%	19.6%
How the analysis population was defined	Treated and meeting the FDA definition (101→96)	Per protocol (107→103)	Reached the three-month assessment	All treated	Treated and reached the 3-month assessment (112→110)
CR definition	At approximately three months	At any time ᵃ	At the three- or six-month assessment	At any time	At any time
Response assessment	Cystoscopy and cytology, central confirmation	Investigator; protocol biopsy at month 12	Investigator ᵇ	Central cytology and biopsy; central pathology at weeks 24 and 48	Central pathology; mapping biopsy at month 12
Re-induction permitted	Not applicable	No	Yes	No	Yes
Data cut-off	25 May 2020	8 July 2019	NR	31 Mar 2025	23 Jun 2025
Median follow-up (months)	36.4	19.7	23.9	20.2 ᶜ	25.8
CR, n/N (%)	39/96 (41)	55/103 (53.4)	58/82 (71)	70/85 (82.4)	83/110 (75.5)
Primary 12-month quantity (%)	18/96 (18.8)	25/103 (24.3)	37/82 (45.1) ᵈ	39/85 (45.9)	51/110 (46.4)
- as reported by the source	18 of 39 responders maintained CR ≥12 months	25 of 103 free of high-grade recurrence at month 12	37 of 82 in CR at the 12-month assessment	CR rate at 12 months, 39 of 85	51 of 110 at the 12-month landmark
- count used here	Re-expressed on the efficacy population	As reported	As reported	As reported	As reported
- anchor	From response	From the first dose	From the first dose	From the first dose	From the first dose
- what the count represents	Achieved CR and maintained it ≥12 months, on the efficacy population	Free of high-grade recurrence at month 12	In CR at the 12-month assessment	In CR at 12 months from the first dose	In CR at the 12-month landmark
Grade ≥3 treatment-related adverse events ᵉ	13/101 (12.9%)	NR	NR	11/85 (12.9%)	0%

**Table 2:** Counts entered into each of the 10 specifications Counts are numerator/denominator. The source of each count, and whether it was reported directly or re-expressed, is given in Table 1. Specifications 2 and 3 differ only in the cretostimogene numerator, because the three-month count has never been published: 69 excludes the 14 complete responses that followed re-induction, and 83 assumes all responses were present at three months. Specification 4 removes responses obtained after re-induction, which affects NAI plus BCG (58 to 45) and cretostimogene (83 to 69) only; the other three trials either did not permit re-induction or recorded all responses within the first induction. Specifications 6 and 10 use the number of patients who received at least one dose as the denominator. Specification 8 substitutes the count reported in the FDA review of the NAI plus BCG cohort, re-expressed on the 77-patient efficacy population used in that review. Each count was modelled with an independent binomial likelihood and a Beta(1,1) prior. Marginal posterior medians and credible intervals were obtained from the Beta quantile function; all simulation-based outputs came from a single set of 100,000 draws under seed 20260809, using R version 4.5.3 (R Foundation for Statistical Computing, Vienna, Austria). CR: complete response; NAI: nogapendekin alfa inbakicept; BCG: Bacillus Calmette-Guérin; FDA: US Food and Drug Administration.

#	Specification	Pembrolizumab	Nadofaragene	NAI + BCG	TAR-200	Cretostimogene
1	CR, each trial's own definition	39/96	55/103	58/82	70/85	83/110
2	CR harmonised to three months, cretostimogene lower bound	39/96	55/103	45/82	67/85	69/110
3	CR harmonised to three months, cretostimogene upper bound	39/96	55/103	45/82	67/85	83/110
4	CR, first-induction responses only	39/96	55/103	45/82	70/85	69/110
5	12-month, counts of Table 1	18/96	25/103	37/82	39/85	51/110
6	12-month, all-treated denominators	18/101	25/107	37/88	39/85	51/112
7	12-month, TAR-200 replaced by 37/85	18/96	25/103	37/82	37/85	51/110
8	12-month, NAI plus BCG replaced by 28/77	18/96	25/103	28/77	39/85	51/110
9	12-month, TAR-200 37/85 and NAI plus BCG 28/77	18/96	25/103	28/77	37/85	51/110
10	12-month, all-treated denominators and TAR-200 37/85	18/101	25/107	37/88	37/85	51/112

Eligibility and search

Prospective trials that (a) enrolled patients with BCG-unresponsive high-risk NMIBC as defined by the FDA [2], (b) reported a cohort containing CIS, (c) reported CR as a primary endpoint, (d) evaluated a regimen that was approved by the FDA for this indication or the subject of a rolling application initiated by 10 August 2026, and (e) reported a 12-month response-related quantity as a count or in a form from which a count could be recovered were included.

Criterion (d) restricts the analysis to the contemporary pivotal cohorts, excluding SWOG S1605 [12] and oportuzumab monatox, for neither of which is an application under review. It also excludes intravesical chemotherapy regimens such as sequential gemcitabine and docetaxel, which are used in practice and carry guideline support [1] but have no regulatory approval for this indication and no prospective registration cohort with CR as the primary endpoint under the FDA definition. These regimens are therefore outside the question posed here, which concerns the ordering among agents that reached regulatory review on such cohorts.

PubMed was searched on 10 August 2026, which returned 394 records. The search combined bladder with the phrases BCG-unresponsive, BCG unresponsive, unresponsive to BCG, Calmette-Guerin-unresponsive, and Calmette-Guérin-unresponsive, all restricted to the title and abstract fields. The five regimens were also enumerated from FDA and other regulatory disclosures, and registry records used to verify counts and definitions.

Endpoints

The two endpoints were CR under each trial’s own primary definition, and a 12-month response-related quantity derived from each trial’s published data. For brevity, the five quantities entered into the second analysis are called the primary 12-month quantities.

The primary CR and 12-month counts were taken from the primary reports of the five trials [3-7]. The five 12-month quantities are not harmonised: they differ in anchor, in analysis population and in what they measure, and one was re-expressed for this analysis. Table 1 gives the source and the count used for each cohort. For pembrolizumab, the source reports 18 of 39 responders maintaining CR for at least 12 months [3], which was re-expressed on the 96-patient efficacy population as 18 of 96. For TAR-200, the published observed CR rate at 12 months was 39 of 85 [6]. A separately reported Kaplan-Meier duration-of-response estimate of 56.2% was not used.

All five cohorts are single-arm trials without a concurrent control, so each carries a high risk of bias for between-agent comparison. Instead of a tool designed for comparative studies, the features most likely to affect the estimate were tabulated (Table 1).

Statistical analysis

Each count was modelled with an independent binomial likelihood and a Beta(1,1) prior. Marginal posterior medians and 95% credible intervals (CrI) came from the Beta quantile function. All simulation-based outputs came from one saved analysis object under seed 20260809, with 100,000 draws per specification.

All 10 pairwise probabilities, the probability that each agent ranks first, and the surface under the cumulative ranking curve (SUCRA) were computed. SUCRA is used only as a descriptive summary of the rank distribution within each specification. No network meta-analysis and no borrowing across cohorts were done. A comparison is called resolved when the 95% CrI for the difference excluded zero, a descriptive convention rather than a hypothesis test.

Specification Grid

More than one defensible analysis can be built from the public record, so 10 specifications were examined rather than choosing one. Four concern CR: each trial’s own definition; harmonisation to three months with the unpublished cretostimogene count at its lower and at its upper bound; and first-induction responses only. Six concern the 12-month quantities: the counts of Table 1; those counts on all-treated denominators; TAR-200 replaced by 37 of 85; NAI plus BCG replaced by 28 of 77; and the two combinations. The counts entered into each specification are given in Table 2. The grid is not exhaustive, and the 10 specifications reuse the same five cohorts, so they are not independent.

Additional Analyses

For each pair, a direction-reversal tipping point was computed: the downward shift applied to the higher cohort’s response probability at which the posterior probability that it exceeds the other reaches 0.5. For a pure location shift, this equals the posterior median of the pairwise difference. It is descriptive, not an estimate of any bias present. One agent, cretostimogene, had not received regulatory approval by the cut-off date and remains under a rolling application. The analyses were therefore repeated with the four approved agents only, to examine whether its inclusion affected the findings. Prior sensitivity was also assessed by repeating the analyses under a Jeffreys prior and under sceptical priors centred on the 20% historical control rate in the FDA guidance [2]. Because these comparisons are unanchored, they cannot be credibly adjusted for between-cohort differences with aggregate data [13].

Analyses used R version 4.5.3 (R Foundation for Statistical Computing, Vienna, Austria). A large language model (Claude, Anthropic, San Francisco, CA) was used to draft and debug the analysis code; the author verified all outputs against the source data and is responsible for the content. The analysis code is available from the author on request.

## Results

Included cohorts

Of 394 records screened, 38 reports were assessed in full, and five cohorts met the criteria. The efficacy populations were 96 for pembrolizumab, 103 for nadofaragene, 82 for NAI plus BCG, 85 for TAR-200 and 110 for cretostimogene. No two used the same combination of response definition, analysis population, assessment schedule and re-induction policy (Table 1). CR was assessed at approximately three months in one trial, at the three- or six-month assessment in one, and at any time in three. Re-induction was permitted in two of the five. Median follow-up ranged from 19.7 to 36.4 months.

Complete response and the 12-month quantities

Estimated CR rates, which are posterior medians and therefore differ slightly from the raw proportions given in Table 1, were 81.9% for TAR-200 (95% CrI 72.9 to 89.0, from 70 of 85), 75.1% for cretostimogene (66.6 to 82.5, from 83 of 110), 70.4% for NAI plus BCG (60.1 to 79.5, from 58 of 82), 53.4% for nadofaragene (43.8 to 62.8, from 55 of 103) and 40.8% for pembrolizumab (31.3 to 50.7, from 39 of 96) (Table 3).

**Table 3:** Posterior estimates and rank probabilities Medians and intervals are analytic; rank probabilities come from the saved analysis object. Percentages given with a credible interval are posterior medians; those given with a count are the observed proportions used. The primary 12-month quantities are not harmonised in anchor, population or construct (Table 1), and the rank probabilities in that column should be read accordingly. CR: complete response; CrI: credible interval; NAI: nogapendekin alfa inbakicept; BCG: bacillus Calmette-Guérin.

Agent	CR (%) (95% CrI)	Probability of ranking first, CR	Primary 12-month quantity (%) (95% CrI)	Probability of ranking first, 12 months
Pembrolizumab	40.8 (31.3–50.7)	<0.001	19.2 (12.2–27.7)	<0.001
Nadofaragene	53.4 (43.8–62.8)	<0.001	24.6 (17.0–33.4)	<0.001
NAI + BCG	70.4 (60.1–79.5)	0.03	45.2 (34.8–55.9)	0.29
TAR-200	81.9 (72.9–89.0)	0.85	45.9 (35.7–56.5)	0.34
Cretostimogene	75.1 (66.6–82.5)	0.12	46.4 (37.3–55.7)	0.37

All six comparisons between the higher three and the lower two excluded zero; none of the four comparisons within these groups did, although two were close (nadofaragene versus pembrolizumab, 0.964; TAR-200 versus NAI plus BCG, 0.961). The probability of ranking first was 0.854 for TAR-200, 0.120 for cretostimogene and 0.026 for NAI plus BCG, and below 0.001 for the other two; the probability of ranking last was 0.964 for pembrolizumab and 0.036 for nadofaragene.

The corresponding primary 12-month quantities were 45.9% for TAR-200 (95% CrI 35.7 to 56.5, from 39 of 85), 46.4% for cretostimogene (37.3 to 55.7, from 51 of 110), 45.2% for NAI plus BCG (34.8 to 55.9, from 37 of 82), 24.6% for nadofaragene (17.0 to 33.4, from 25 of 103) and 19.2% for pembrolizumab (12.2 to 27.7, from 18 of 96) (Figure 1). These are not the same quantity measured again: they differ from the CR estimates, and from one another, in anchor, population and construct (Table 1).

**Figure 1:**
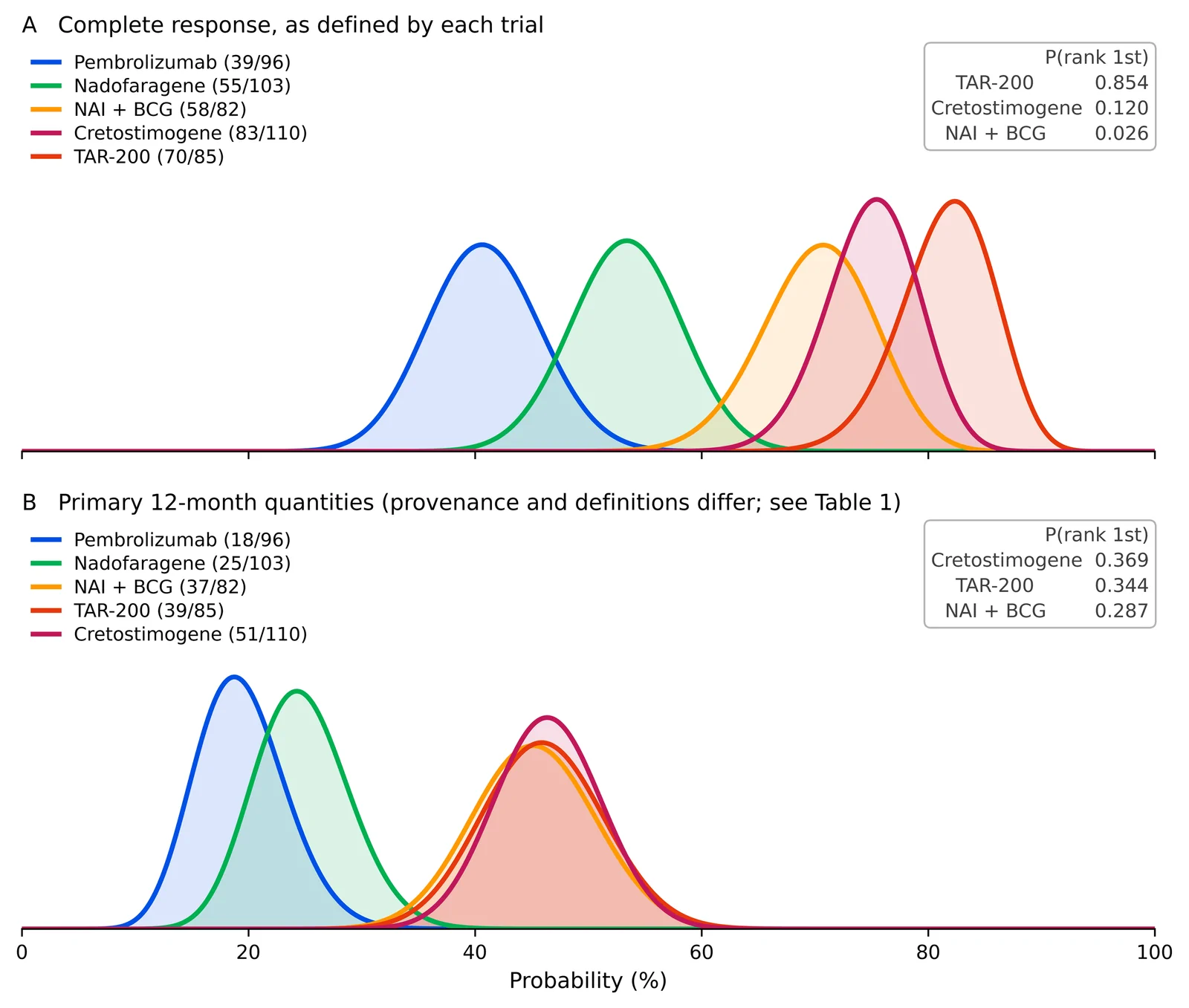
Posterior distributions for complete response and for the primary 12-month quantities (A) Complete response as defined by each trial. (B) The primary 12-month quantities, which differ in anchor, population and construct and are shown together for reference rather than as a harmonised comparison (Table 1). Curves are Beta posterior densities from the counts shown in the legend. Colours identify the agents in both panels. Boxes give the probability that each agent ranks first; the leading agent differs between panels. Figure created in R version 4.5.3 (R Foundation for Statistical Computing, Vienna, Austria).

The two groupings are apparent in both, while the spread within the higher three is much smaller at 12 months. All six comparisons between the higher three and the lower two excluded zero. The differences were 25.9 percentage points for NAI plus BCG against pembrolizumab (95% CrI 12.6 to 38.8), 20.5 against nadofaragene (6.9 to 33.7), 26.6 for TAR-200 against pembrolizumab (13.4 to 39.4), 21.3 against nadofaragene (7.8 to 34.4), 27.2 for cretostimogene against pembrolizumab (14.8 to 38.9) and 21.8 against nadofaragene (9.1 to 33.9). The smallest was NAI plus BCG against nadofaragene.

Within the higher group, the three estimates lie within 1.5 percentage points of one another. The differences were -0.8 percentage points for NAI plus BCG versus TAR-200 (95% CrI -15.5 to 14.2), -1.2 for NAI plus BCG versus cretostimogene (-15.1 to 12.9) and -0.5 for TAR-200 versus cretostimogene (-14.3 to 13.4), with pairwise probabilities of 0.460, 0.432 and 0.473. The probability of ranking first was 0.369 for cretostimogene, 0.344 for TAR-200 and 0.287 for NAI plus BCG, and the probability of ranking last was 0.824 for pembrolizumab and 0.176 for nadofaragene. Numerical similarity is not equivalence: the posterior range across the three, the maximum minus the minimum within each draw, had a median of 8.3 percentage points (95% CrI 1.6 to 18.9).

Sensitivity to response definition and analysis choices

Harmonising CR to the three-month assessment moved the estimate for NAI plus BCG down by 15.6 percentage points and cretostimogene by up to 12.6. TAR-200 moved by 3.5, and the other two did not move. Under the trial-specific definitions the difference between the two highest CR estimates was 6.7 points. The cretostimogene three-month count has not been published. The harmonised probability that TAR-200 ranks first is therefore not a single value: 0.99 at the lower end of the plausible range and 0.70 at the upper end. Counting only first-induction responses removed a clean separation between the two groups: the probability that NAI plus BCG exceeds nadofaragene fell from 0.99 to 0.58.

Substituting alternative 12-month quantities left TAR-200 and cretostimogene unresolved in every case, but changed the position of NAI plus BCG. The trial publication reports 37 of 82 [5]; the FDA review reports 28 of 48 responders maintaining CR for at least 12 months, which was re-expressed as 28 of 77 [11]. The two differ at once in population, anchor and who assessed response.

How much of the ranking survives

Figure 2 shows all 10 pairwise comparisons under the 10 specifications. Three were resolved in all ten: pembrolizumab below TAR-200, pembrolizumab below cretostimogene, and nadofaragene below TAR-200. One claim, nadofaragene above pembrolizumab, held in none, although its direction was consistent throughout, with the probability ranging from 0.82 to 0.96. The remaining six held in some specifications and not others: cretostimogene above nadofaragene in eight of ten, NAI plus BCG above pembrolizumab in seven, NAI plus BCG above nadofaragene in five, TAR-200 above NAI plus BCG in three, TAR-200 above cretostimogene in two, and cretostimogene above NAI plus BCG in one. Of the 100 cells, 44 were unresolved and none was resolved in opposite directions; among the 56 that resolved, TAR-200 was the higher agent in 25, cretostimogene in 19, and the lower two in none. Because the 10 specifications reuse the same five cohorts, these counts describe the sensitivity of each claim to the analytic choices examined; they are not independent replications.

**Figure 2:**
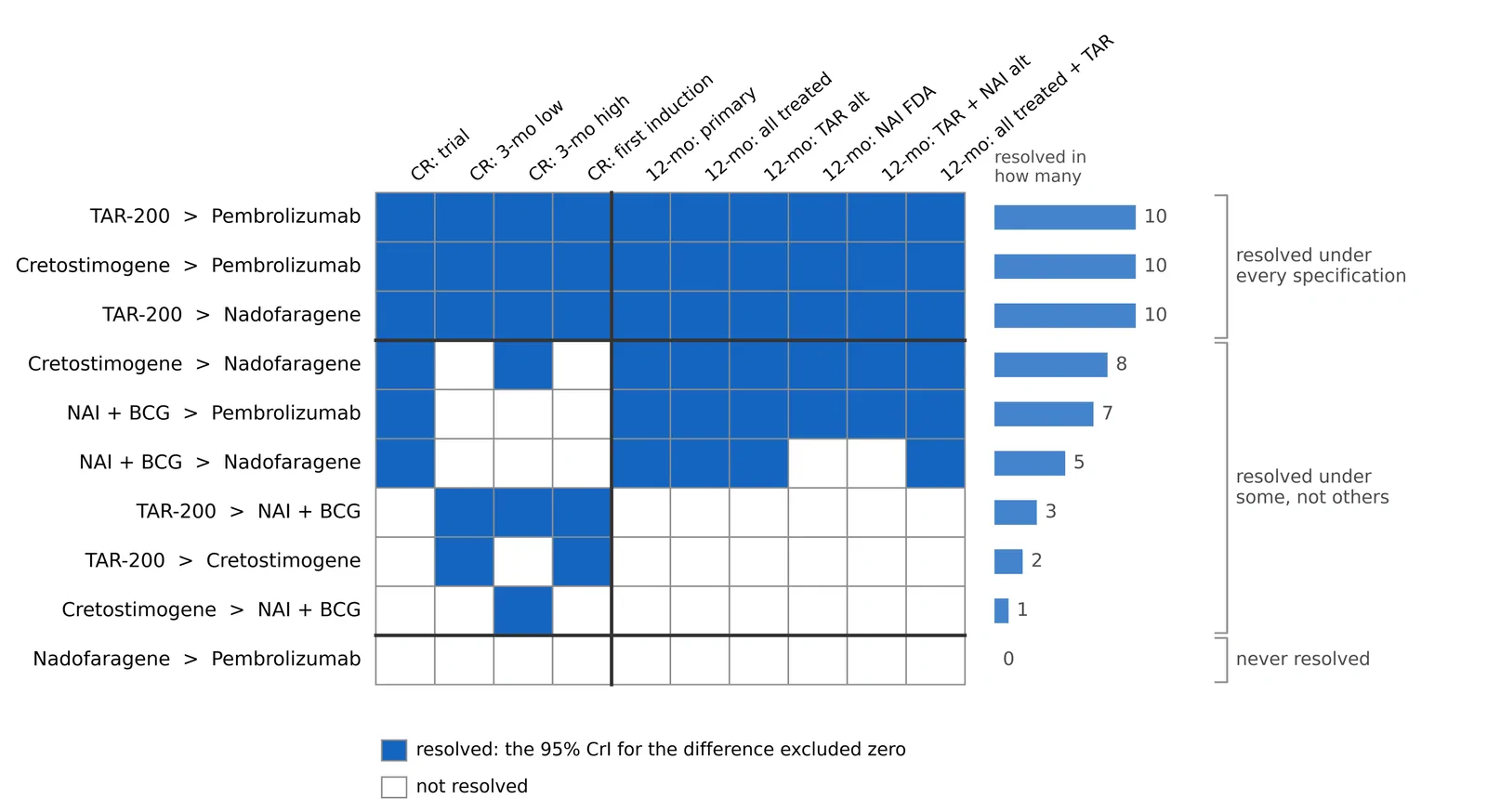
All pairwise comparisons under the 10 specifications Each row is a directional claim about one pair; each column is one of the 10 specifications, four for complete response and six for the 12-month quantities, all defined in Methods. A filled cell means the 95% credible interval for the difference excluded zero in that specification. No claim was resolved in the opposite direction in any specification, so the direction is stated once in the row label. Rows are ordered by the number of specifications in which the claim was resolved, shown at the right. Forty-four of the 100 cells are unresolved. The grid is not exhaustive and the specifications are not independent of one another (Methods). Figure created in R version 4.5.3 (R Foundation for Statistical Computing, Vienna, Austria).

For NAI plus BCG, the unresolved comparisons with the lower two agents were concentrated in specifications that either excluded responses obtained after re-induction or substituted the FDA-derived count. These were also the specifications that moved its estimate furthest downward, from 70.4% to 54.8% for CR and from 45.2% to 36.6% at 12 months.

Across specifications, pembrolizumab and nadofaragene remained lower, whereas TAR-200 and cretostimogene overlapped substantially (Figure 3A). The SUCRA ranges for pembrolizumab (0.01 to 0.05), nadofaragene (0.21 to 0.37) and NAI plus BCG (0.39 to 0.75) do not overlap one another, whereas those for TAR-200 (0.69 to 1.00) and cretostimogene (0.70 to 0.89) overlap over most of their length. The probability that TAR-200 ranks first moves between 0.23 and 1.00 with the specification. None of the three comparisons within the upper group was resolved under any 12-month specification; they separated only in CR specifications that depend on an unpublished count or on removing re-induction responses.

**Figure 3:**
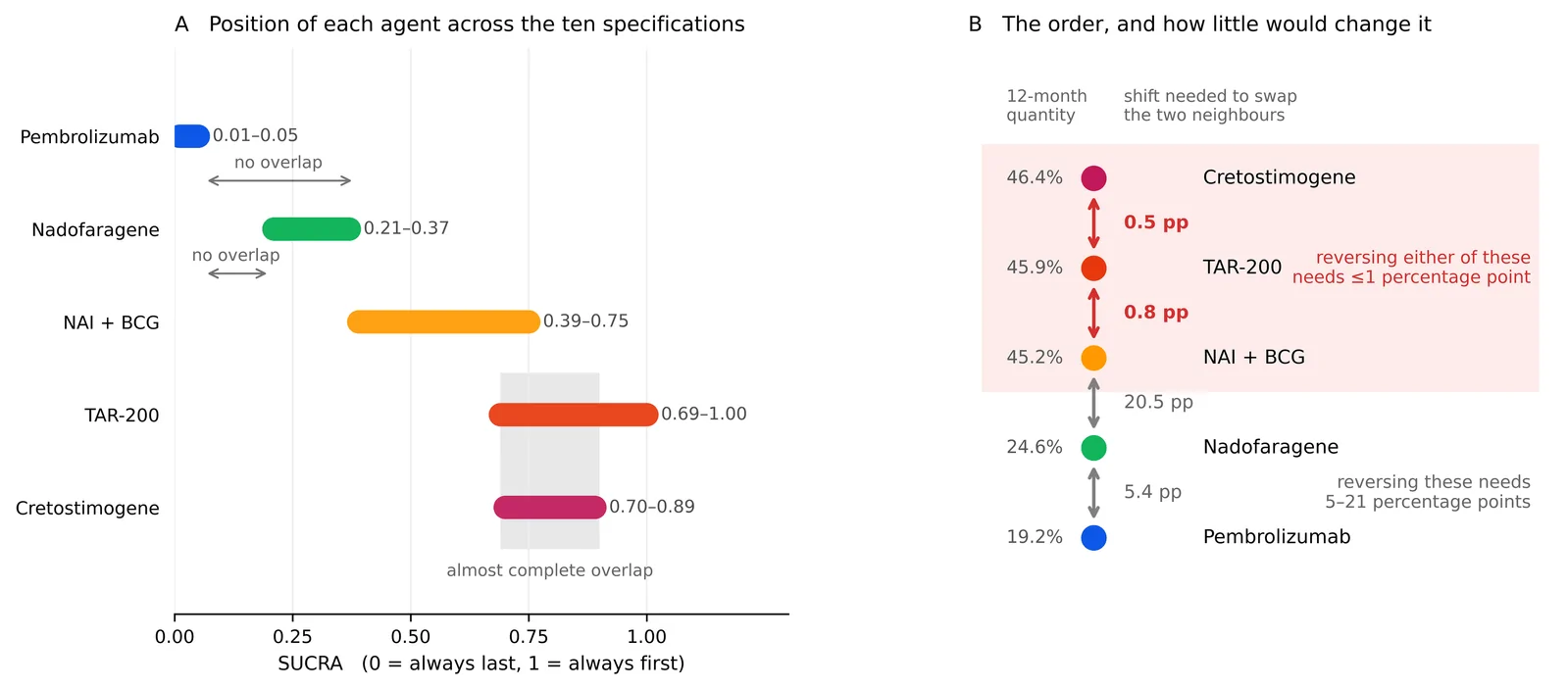
SUCRA ranges and tipping points for the five agents (A) Range of SUCRA for each agent across the 10 specifications, on a 0 to 1 scale where 1 denotes always first. The ranges for pembrolizumab, nadofaragene and NAI plus BCG do not overlap; those for TAR-200 and cretostimogene almost coincide. (B) The five agents in the order of their primary 12-month quantities, with the direction-reversal tipping point for each adjacent pair, that is, the downward shift applied to the higher quantity at which the posterior probability that it exceeds the lower reaches 0.5. This equals the posterior median of the pairwise difference. For comparison, shifts of 12.6 to 15.6 percentage points were observed when CR was harmonised to three months. Neither panel is a test of comparative efficacy. SUCRA: surface under the cumulative ranking curve; CR: complete response; NAI: nogapendekin alfa inbakicept; BCG: bacillus Calmette-Guérin; pp: percentage points. Figure created in R version 4.5.3 (R Foundation for Statistical Computing, Vienna, Austria).

For the primary 12-month quantities, the order of cretostimogene and TAR-200 would reverse if the higher quantity were lower by 0.5 percentage points. For TAR-200 and NAI plus BCG the figure is 0.8 points, and for nadofaragene and pembrolizumab 5.4 points (Figure 3B). Because the tipping point equals the posterior median of the pairwise difference, the figures for the comparisons between the groups are the differences given above, 20.5 to 27.2 points. These are larger than any shift observed here, including the 12.6 to 15.6 points produced by harmonising the CR assessment. On the CR estimates the corresponding figures within the higher group are 4.7 points for cretostimogene versus NAI plus BCG, 6.7 for TAR-200 versus cretostimogene and 11.4 for TAR-200 versus NAI plus BCG.

Analysis restricted to the approved agents

Repeating the analyses with the four approved agents only left the findings unchanged. At 12 months the two higher agents remained separated from the two lower agents in all four comparisons, by 20.5 to 26.6 percentage points, against 20.5 to 27.2 points when cretostimogene was included. NAI plus BCG and TAR-200 remained unresolved at 12 months (probability 0.460; tipping point 0.8 percentage points, the same value as in the five-agent analysis) and separated in three of the ten specifications, all of them CR specifications. Of the 60 cells in the reduced grid, 25 were unresolved and none was resolved in opposite directions.

Prior sensitivity

A Jeffreys prior moved estimates by no more than 0.4 percentage points, and sceptical priors centred on 20% left the ordering unchanged although posterior medians moved by up to 11.3 points.

## Discussion

Principal findings

These findings apply to BCG-unresponsive NMIBC with CIS, not to papillary-only disease.

The clinical interpretation is straightforward. The published quantities form an upper and a lower group rather than a ranking of five. At 12 months, TAR-200, cretostimogene and NAI plus BCG were each about 45%, against 25% and 19% for the other two, every comparison between the groups differing by 20 to 27 percentage points. None of the three comparisons within the upper group was resolved under any 12-month specification.

The boundary is not equally secure along its length. The comparisons involving TAR-200 and cretostimogene held in almost every specification, whereas NAI plus BCG was above the lower two in 12 of 20 cells. Its estimate is an upper-group estimate, but its separation from the lower group moves most under the specifications that alter response attribution or the reported 12-month construct. This pattern shows that the apparent separation of NAI plus BCG from the lower group is particularly sensitive to response attribution and to the definition of the 12-month quantity.

Neither group is ordered internally. Pembrolizumab and nadofaragene could not be separated from each other, and within the upper group the order would reverse if the higher quantity were lower by about one percentage point. The contrast with the between-group comparisons is instructive: those would need 20 to 27 percentage points to reverse. The largest numerical shift in these sensitivity analyses arose when the CR assessment was harmonised to three months. It moved individual estimates by up to 15.6 percentage points, more than the difference between the two highest reported rates but far less than the smallest 12-month gap between the groups. Before two published rates are compared, four things about each should be checked: how response was defined, how the denominator was built, when the clock started, and whether re-induction responses were counted (Table 1). These are properties of quantities derived from published data under stated specifications, not estimates of comparative efficacy.

Heterogeneity of reporting

The heterogeneity is not confined to the definition of response. No two cohorts built the analysis population by the same rule. The consequence is visible in the regulatory record: reviewing the same NAI plus BCG cohort, the FDA used a 77-patient population and reported a CR rate of 62%, against 71% in the trial publication [5,11]. A smaller difference is present for TAR-200: the trial publication analysed 85 treated patients [6], whereas the FDA approval announcement describes the pivotal cohort as enrolling 83 patients [14]. The cretostimogene trial has not published a three-month response rate [7], so the simplest like-for-like comparison cannot be completed from the public record. None of this implies error; each trial reported its own results correctly, and the difficulty arises only when the figures are taken out of their trials. It cannot be said that reporting choices rather than the agents account for the differences, because the two are not separable here.

The present findings are consistent with the existing literature. Published syntheses and commentaries have placed bladder-sparing regimens side by side across trials while acknowledging differences in study design and endpoints [9,10]. Neither examined how far the apparent ordering depends on those differences, which is what the present analysis adds. The heterogeneity documented here persists despite earlier recommendations from the International Bladder Cancer Group for standardised definitions and endpoints in trials of non-muscle-invasive bladder cancer [15]. Because these data do not establish comparative efficacy within the upper group, treatment burden, route and frequency of administration, safety, cost and availability remain relevant to the choice between the two approved agents in the upper group and would also apply to cretostimogene if it is approved. This is consistent with the group's recommendation that selection should rest on such factors rather than on a ranking of response rates [16].

Limitations

These are unanchored comparisons: population adjustment would require all prognostic factors and effect modifiers to be adjusted for, which aggregate data do not permit [13]. The cohorts are small, all single-arm, and none designed for comparison; the safety data are not comparable either (Table 1).

Four of the five agents hold FDA approval for this indication; cretostimogene does not and remains under a rolling application, so its inclusion rests on the regulatory milestone defined in criterion (d) rather than on approval. An analysis restricted to the four approved agents gave the same two-group structure and the same failure to order the upper agents. Other agents at a comparable stage of development, such as detalimogene voraplasmid, had not initiated a regulatory submission by the cut-off date and did not report a 12-month count in a form from which a numerator and denominator could be recovered [17]; they therefore did not meet criteria (d) and (e). The boundary drawn here is a regulatory one and will move as further agents reach submission.

The 10 specifications are neither exhaustive nor independent, since they reuse the same five cohorts and often differ by a single choice. Counts such as 56 of 100 cells therefore describe the examined grid and carry no frequentist interpretation, and the grid and several additional analyses were added after the comparative outputs had been examined. The search was limited to PubMed, and one investigator carried out selection, extraction and analysis without independent duplication, which is a potential source of bias.

Finally, the inability to order the three higher agents does not imply that they are equivalent: the posterior range across them extends to 18.9 percentage points, so a clinically meaningful difference cannot be excluded.

## Conclusions

The published response quantities form an upper and a lower group rather than a stable ranking of five. TAR-200 and cretostimogene remained above the lower two agents in most specifications, whereas the separation of NAI plus BCG was less stable. Within the upper group, the agents could not be ordered, and the two specifications that would separate them at three months rest on a cretostimogene count that has not been published.

A single common quantity would make the comparison more secure. Future trials should report, in addition to their own primary definition, the proportion of treated patients free of high-risk disease at 12 months from the first dose. All five trials already report something close, so what is proposed is harmonisation of an existing quantity rather than a new endpoint.
